# A Hybrid Speech Enhancement Algorithm for Voice Assistance Application

**DOI:** 10.3390/s21217025

**Published:** 2021-10-23

**Authors:** Jenifa Gnanamanickam, Yuvaraj Natarajan, Sri Preethaa K. R.

**Affiliations:** 1 Department of Artificial Intelligence and Data Science, KPR Institute of Engineering and Technology, Coimbatore 641407, India; k.r.sripreethaa@kpriet.ac.in; 2 Department of Computer Science and Engineering, KPR Institute of Engineering and Technology, Coimbatore 641407, India; yuvaraj.n@kpriet.ac.in

**Keywords:** speech recognition, speech enhancement, speech to text, word error rate

## Abstract

In recent years, speech recognition technology has become a more common notion. Speech quality and intelligibility are critical for the convenience and accuracy of information transmission in speech recognition. The speech processing systems used to converse or store speech are usually designed for an environment without any background noise. However, in a real-world atmosphere, background intervention in the form of background noise and channel noise drastically reduces the performance of speech recognition systems, resulting in imprecise information transfer and exhausting the listener. When communication systems’ input or output signals are affected by noise, speech enhancement techniques try to improve their performance. To ensure the correctness of the text produced from speech, it is necessary to reduce the external noises involved in the speech audio. Reducing the external noise in audio is difficult as the speech can be of single, continuous or spontaneous words. In automatic speech recognition, there are various typical speech enhancement algorithms available that have gained considerable attention. However, these enhancement algorithms work well in simple and continuous audio signals only. Thus, in this study, a hybridized speech recognition algorithm to enhance the speech recognition accuracy is proposed. Non-linear spectral subtraction, a well-known speech enhancement algorithm, is optimized with the Hidden Markov Model and tested with 6660 medical speech transcription audio files and 1440 Ryerson Audio-Visual Database of Emotional Speech and Song (RAVDESS) audio files. The performance of the proposed model is compared with those of various typical speech enhancement algorithms, such as iterative signal enhancement algorithm, subspace-based speech enhancement, and non-linear spectral subtraction. The proposed cascaded hybrid algorithm was found to achieve a minimum word error rate of 9.5% and 7.6% for medical speech and RAVDESS speech, respectively. The cascading of the speech enhancement and speech-to-text conversion architectures results in higher accuracy for enhanced speech recognition. The evaluation results confirm the incorporation of the proposed method with real-time automatic speech recognition medical applications where the complexity of terms involved is high.

## 1. Introduction

Speech-to-text transcription has gained importance in many applications and benefits in research, the military, medical sector, smart homes, transportation systems, automatic transcription on lectures, conversations, record-making [1]. Speech recognition technology (SRT) involves the identification of patterns in audio waves and matching them with phonetics of speech to convert them into text. The accuracy of SRT dramatically depends on the quality of audio. The presence of background noises, multiple speakers, or the speaker’s accent provides erroneous transcription. Speech enhancement is a significant problem in communications at airports, medical centers, and other familiar public places. The SRT requires enhancement of speech to improve the quality and intelligibility of the signal before translation. Various approaches have been proposed for improving the quality of speech, such as the spectral subtraction algorithm [2], the signal subspace system [3], and the adaptive wiener filtering approach [4].

The spectral subtraction algorithm extracts the speech from additive noise. Enhancement of speech was achieved by estimating the spectrum of the noise-free signal and subtracting the estimated noisy signal from an available observed signal. The spectral subtraction algorithm suffered from residual noise [2]. The signal subspace algorithm was used for enhancing uncorrelated additive noise. This approach decomposes the noisy signal’s vector space into signal plus subspace and orthogonal noise subspace using Karhunen–Loeve transforms (KLT) or eigenvalue decomposition. The signal plus slot is used for processing, and the noisy subspace is discarded [3]. The noisy speech frames were classified into speech-dominated frames and noise-dominated frames using a signal KLT-based technique [4]. A Weiner filter-based algorithm was proposed for enhancing the signals, and it had a drawback of fixed frequency that required estimation of the frequency spectrum of both clean signal and noise before filtering [5]. An Adaptive Wiener filter method was proposed to overcome the disadvantage of the traditional Weiner filer that used an adaptation of the filter transfer function on sample to sample based on speech signal statistics [6]. The adaptive Weiner Filter-based approach was found to provide the best improvement over the signal-to-noise ratio.

The neural networks that learn the statistical information automatically using non-linear processing units were introduced for noise reduction. The deeper networks are considered to be more efficient in learning than the shallow networks [7]. A deep auto-encoder (DAE) algorithm was proposed for training the deep network architectures [8]. The challenge with DAE is the difficulty in generalizing the algorithm for all types of speech signals. Over conventional minimum mean square error (MMSE) based statistical techniques, supervised methods using deep neural networks were proposed to enhance the large volume of speech data. These methods were found to handle non-stationary noises effectively [9]. A voice activity detector (VAD) was introduced to estimate the noise during the non-speech, but it failed on encrypted speech signals [10]. Recurrent neural network (RNN) based speech enhancement techniques were introduced. The RNNs are found to produce significant performance by taking advantage of the temporal information over the noisy spectrum. Long Short-Term Memory (LSTM) was implemented for optimal speech enhancement and produced optimal results [11].

Generative adversarial networks (GAN) were used to construct the clear speech signals from the noise signals over RNNs [12]. Multiple deep neural networks were recommended over the single neural network for speech enhancement with known and unknown sources of noise [13]. A de-reverberation method combining correlation-based blind de-convolution and modified spectral subtraction was presented for speech enhancement where inverse filtered reverberation was suppressed by the spectral subtraction [14]. Though the conventional subtraction method reduces the noise level from speech, it introduces distortion noise in considerable spectral variation. The multiband spectral subtraction algorithm was proposed to overcome the distortion, maintaining the quality of speech signal [15,16]. Recent studies focus on the non-linear spectral subtraction algorithm for speech enhancements due to the significant variation in signal-to-noise ratio. The spectrum of real-world noise is irregular, and they have been affected more adversely at some frequencies. Non-linear spectral subtraction approaches are recommended to handle cleaning the speech signal [17,18]. The sub-space-based signal estimation method was proposed based on modified singular value decomposition (SVD) of data matrices that recovers speech signals from noisy observations [19]. The technique focused on mapping the observed signal to a clean signal, suppressing the noise subspace. Though the subspace-based speech enhancement deals with noise distortion, it is also used to remove colored noise [20].

Generalized sidelobe canceller (GSC) has been used for non-stationary broadband signals. GSC separates the desired signals from the interference by exploiting spatial information about the source location [21]. Noise suppression techniques play a vital role in automatic voice recognition (AVR) strategies, aiming to provide a clear gap between the clean and noisy speech signals [22]. Wiener filter and spectral subtraction combined noise estimator was proposed to control the noise energy in current frames and estimate noise from preceding frames by minimizing over subtraction [23,24,25]. Iterative signal enhancement (ISE) algorithm based on truncated SVD was proposed to obtain improved selective frequency for filtering noises from speech signals. ISE performed better than other classical algorithms, especially with speech signals [26,27].

From the literature study, it is evident that Nonlinear Spectral Subtraction (NSS) and Iterative Signal Enhancement algorithm (ISE) are the most effective methods for speech enhancement [28]. This paper proposes hybridization of NSS and ISE methods for further enhancing the speech signals. The performance of the hybrid algorithm is compared with the implementation of individual practices.

Dynamic time warping (DTW) is a dynamic programming algorithm technique used for determining the correspondence between two sequences of speech that may differ in time or speed [29]. For example, resemblances in speaking a specific word would be identified, even if in one audio the person was speaking slowly and if in another the same person was talking more quickly, or even if there were hastening and slowing down during the course of one observation [30]. DTW can be applied to audio, video, or any data that can be represented using a linear representation. Table 1 gives the insights about the literature survey.

## 2. Proposed Methodology

**Figure 1:**
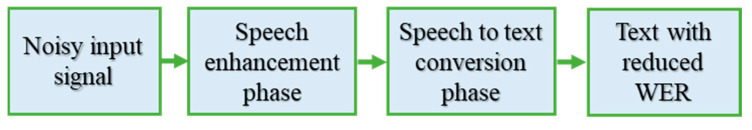
Proposed idea.

This work aims to enhance the speech signal by suppressing the noise signals involved in voice control applications. Speech enhancement is an essential factor in speech recognition as it can be used as a pre-processor to enhance speech. Generally, the source of the noise signals can be background noise, electromagnetically induced noise. Reducing these noises will result in increasing the intelligibility of the speech and agreeable speech recognition. Figure 1 depicts the flow of the idea of enhancing the noisy speech signal.

In this article, a hybridized speech enhancement algorithm is proposed with experimental results. Section 2.1 and Section 2.2 present various speech enhancement and speech-to-text conversion algorithms used for various speech signals, respectively. Section 3 represents the results obtained for the speech enhancement algorithms and their comparisons by using the word error rate values by converting the enhanced speech to text.

### 2.1. Speech Enhancement Algorithms

Enhancement of speech is essential when the terms involved are complex and involve external noises. There are various speech enhancement (SE) algorithms available, which are discussed in the following section.

#### 2.1.1. Iterative Signal Enhancement Algorithm (ISE)

The iterative signal enhancement algorithm is used for reducing the noise in speech. The algorithm is designed based on a trimmed singular value decomposition (SVD) procedure and can be used as a tool for enhancing the noisy speech signal by suppressing the additive noise present in the signal. Compared to the classical algorithms, the ISE algorithm escalates similar improvements in Signal-to-Noise Ratio (SNR).

The ISE algorithm executes in two phases. The first phase produces an enhanced signal s[i] from the noisy signal ns[i]. The enhanced signal s[i], which comes from phase I, still contains some noise. So, the noise removal phase is repeated a certain number of times depending on the level of noise present in the signal. The signal decomposition of the enhanced signal at each level is given a rank ranging from 1 to level l. The first rank signal decomposition sd[i] for the s[i] is obtained by averaging the anti-diagonals of the Hankel matrix to rank-1. Hankel matrices are square matrices with constant ascending skew-diagonals from left to right. Hankel matrix is constructed from the signal is helpful for decomposition of constant signals and time-frequency representation. The rank-1 signal decomposition covers most of the energetic spectral band of the noisy input signal in the frequency domain.

**Figure 2:**
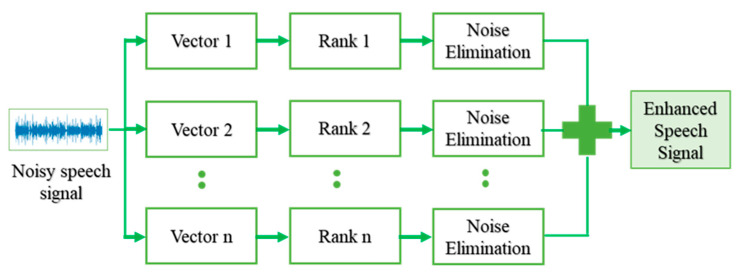
Iterative Signal Enhancement Algorithm.

The signal decomposition sd[i] is subtracted from the input signal sn[i] to calculate the residual signal r[i], and the phase-I procedure is repeated by using the residual signal as the input for the upcoming iterations. The signal decomposition sd[i] is summed up throughout the iterations to obtain the enhanced signal s[i]. The iteration is stopped once the residual signal r[i] contains only the noise components. The phase-2 concentrates on the residual signal to maintain the number of iterations of phase-I. The working of the ISE algorithm is depicted in Figure 2.

The ISE algorithm is advantageous over the typical enhancement algorithms as ISE has a better frequency selectivity for filtering out the noise than the standard algorithms.

#### 2.1.2. Subspace—Based Speech Enhancement

Subspace methods, also called high-resolution or super-resolution methods, use an eigenvalue analysis or eigendecomposition of the correlation matrix to derive frequency component values of a signal. The process for subspace-based speech enhancement can be stated as follows:

(1) Isolating the subspace as signal and noise subspaces from the original subspace
  (noise mixed speech)
(2) Eliminating the noise-only subspace that has been isolated in step1.

Assume *s(i)* represent the pure speech signals and let *n(i)* indicates the zero-mean additive noises mixed in the pure speech. The observed noisy speech *x(i)* can be given by

*x(i) = s(i) + n(i)*

Allow Rx, Rs, and Rn to be (*p* × q) genuine autocorrelation matrices of *x(i), s(i)*, and *n(i)*, respectively, with q > *p*. Rx = Rs + Rn is obvious given the assumption of uncorrelated speech and noise. Regardless of the specific optimization criterion, speech enrichment is now obtained by

1. By nullifying the components in the noise subspace, the enhanced speech is constrained to inhabit only the signal subspace.
2. Changing (decreasing) the eigenvalues of the signal subspace.

**Figure 3:**
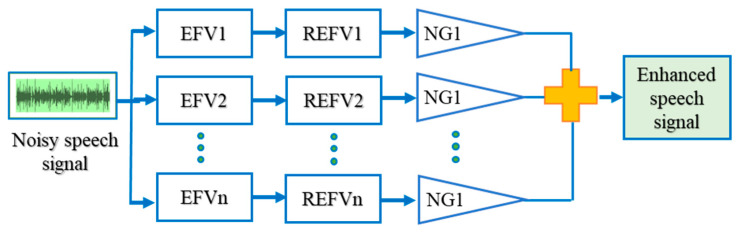
Subspace—based speech enhancement.

The input speech signal with noise is split into unique spaces. Each and every space is individually processed. In Figure 3, the noisy input signal is fed as the input, whereas the individual subspaces are allocated with a vector value and rank, respectively. The filter bank output of each subspace is obtained by filtering the noisy signal *n(i)* with its corresponding eigenfilter vector (EVF) and its reversed eigenfilter vector (REFV). This filtered output is fed to the not gate (NG) and their summation results in the enhanced speech signal.

#### 2.1.3. Nonlinear Spectral Subtraction (NSS)

**Figure 4:**
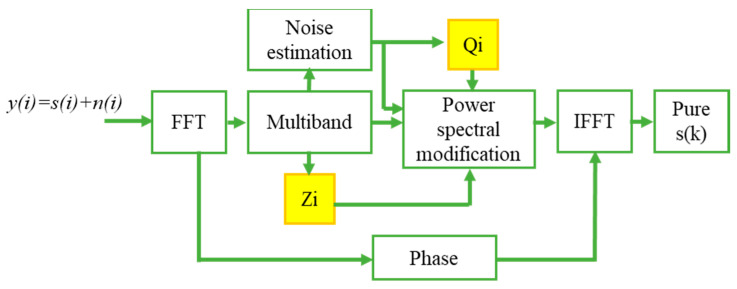
Nonlinear spectral subtraction.

Nonlinear Spectral subtraction is one of the most primitive and notably the most famous speech enhancement methods. NSS can be predominantly valid in cases where a boisterous environment contaminates an original speech signal with the same bandwidth as that of speech. To decrease external noise, the NSS algorithm is developed, which considers the change of signal-to-noise ratio across the speech spectrum and uses a distinct over-subtraction factor in each frequency band. Figure 4 describes the steps involved in extracting only the speech from the noisy speech signal. The speech signal with noise is given as input to the Fast Fourier Transform module. Let *y(i)* denote the noisy speech, i.e., the pure speech signal *s(i)* is polluted with the noise signal *n(i)*. Then in the time domain, their relationship is described as:

*y(i) = s(i) + n(i)* (1)

To obtain their relation in the spectral domain, take the Discrete Fourier Transform (DFT) and power magnitude for Equation (1) with the assumption that noise and speech signals are uncorrelated, then the relation is described as follows:

|Y(r,f)|^2^ = |S(r,f)|^2^ + |N(r,f)|^2^ (2)

where r and f denote the frame and frequency values respectively. Now with the assumption that |N(r,f)| and |$\hat{N}$(r,f)| can be estimated, the spectral subtraction can be formulated as follows:

(3) $$|\hat{S}(r,f)|{}^{2}=|Y(r,f)|{}^{2}-|\hat{N}(r,f)|{}^{2}$$

The spectral subtraction algorithm adapts the damaged speech signal’s short-term spectral magnitude appropriately. The signal is modified so that the synthesized signal feels as near to the unbroken voice signal as possible. A noise power estimate and a subtraction rule are used to calculate the appropriate weighting of spectral magnitudes.

The Word Error Rate represents the amount of word error occurring during the speech. It can be calculated using the formula

WER = Ns + Nd + (Ni/Nn)(4)

where ‘Ns’ represents the number of substitutions, ‘Nd’ indicates the number of deletions, ‘Ni’ means the number of insertions, and ‘Nn’ stands for the number of words in a sentence.

### 2.2. Speech to Text Conversion

The primary purpose of speech to text (STT) or speech recognition is to enable a real-time dictation of audio signals into text. All the STT systems depend on the acoustic model and the language model. In the case of including the additional feature vocabulary systems, a pronunciation model can be used. It is challenging to construct a universal speech recognizer. To develop the best quality STT system, it needs to specialize in a specific language, idiom, application domain, speech type, and communication frequency.

#### Hidden Markov Model

Hidden Markov Models (HMM) are mainly used for general-purpose speech recognition systems. In general, the speech signals are observed as a stationary signal whose amplitude and frequency remains constant. It takes a very short time scale for a speech to be estimated as a static process.

In HMM, it is possible to train the data set automatically, which makes it easy computationally, and hence it is extensively used. The HMM would generate a sequence of n-dimensional real-valued vectors every ten milliseconds (with ‘n’ being a small integer value such as 5 or 10) in speech recognition. The first coefficient of a Fourier transform of a small part of the speech is extracted and decorrelated with the cosine transform to calculate the cepstral coefficient vectors.

In each state of the HMM, a statistical distribution of a mixture of diagonal covariance Gaussians is performed to present the probability of each identified vector. In the current speech recognition systems, every word or phoneme has its output distribution, whereas, in HMM, the sequence of the terms or phonemes is constructed by cascading the individually trained cepstral vectors for the specific words and phonemes, respectively.

Modern voice recognition systems use diverse combinations of various common strategies to improve outcomes beyond the fundamental approach. Context dependency of the phonemes is required for the traditional large-sized vocabulary system to enable the phonemes with different pre and post contexts to have unique HMM states. These HMM states can be used to normalize different recordings and the speaker conditions using the cepstral normalization method. Vocal tract length normalization (VLTN) can be used to further normalize male-female or other speaker criteria. To capture the speech dynamics, linking and linear discriminant analysis (LDA) based projects can be used, which is followed by either the heteroscedastic LDA step or global semi-tied covariance transform method. To optimize the classification-related measure of the training data, many systems employ discriminative training strategies, which can be used to avoid the purely statistical approach to estimate the HMM parameter.

**Figure 5:**
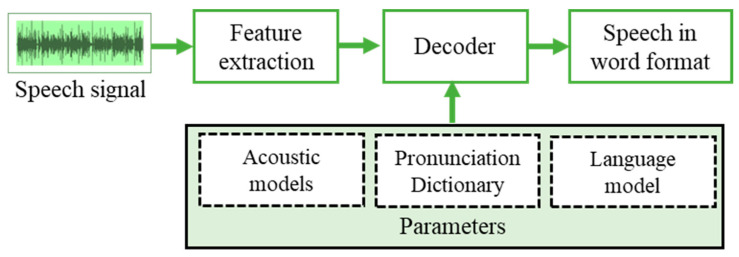
Working model of Hidden Markov Model.

The HMM is used to convert speech features into HMM parameters and calculate all speech samples’ likelihood. Recognition of this likelihood of speech samples is used to recognize the spoken words. Figure 5 represents the working model of the HMM that decodes the extracted features based on the parameters such as acoustic models, pronunciation dictionary, and the language model for which speech recognition is required.

## 3. Hybrid Speech Enhancement Algorithm (HSEA)

The speech signal can be a single, continuous, or spontaneous word. The spontaneous words are complicated as their speech is fast and the number of words is high. This spontaneousness creates complications in automatic speech recognition. The proposed work mainly concentrates on spontaneous speech in the medical domain. The words used in the medical environment are complicated, and the medication prescriptions need clarity. Hence, the idea is to develop a hybridized algorithm that can resolve the issue and assure accuracy.

**Figure 6:**
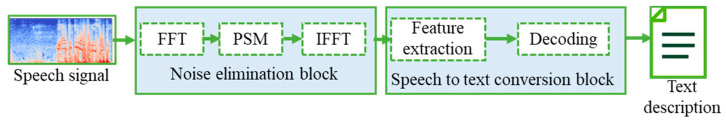
Proposed hybridized algorithm for speech enhancement.

The nonlinear spectral subtraction and the hidden Markov model are cascaded to form a hybrid architecture to enhance the accuracy of the speech recognition system. The NSS provides a better result for the spontaneous signal by suppressing its noise to a greater extent, and the HMM can give high accuracy in converting the speech to text. Figure 6 shows the proposed hybrid algorithm architecture to enhance the speech signal by cascading the NSS and the HMM algorithms. The steps involved in the HSEA is described in Algorithm 1.
**Algorithm 1** Hybrid Speech Enhancement Algorithm**Input** *y(i)* the noisy speech signal with *n(i)* the noise and *s(i)* the clear speech signal
**Segmentation:** the speech signal is segmented into frames.**Process:** The noisy signal is processed frame by frame and the Fourier transform is calculated as y(r,f) = s(r,f) + *n*(r,f)where ‘r’ is the frame number.**Calculation:** The short-term power spectrum of *y(i)* is calculated as |y(f)|^2^ = |s(f)|^2^ + |*n*(f)|^2^**Estimation:** By removing the noise from the input signal, the speech is estimated as follows: $|\hat{s}(f){|}^{2}=|y(f){|}^{2}-|\hat{n}$(r,f)|^2^
**Obtain:**$\mathrm{The}\;\mathrm{noise}\;\mathrm{spectrum}\;|\hat{n}$(f,t)|^2^ is obtained by averaging the recent pause frames $\;|\hat{n}$$(r,f){|}^{2}=\frac{1}{N}\sum_{k=0}^{N-1}\left|yk\left(f\right)\right|$^2^where N is the number of consecutive speech frames**Reconstruction:**$\;\mathrm{Step}\;5\;\mathrm{is}\;\mathrm{reconstructed}\;\mathrm{by}\;\mathrm{expressing}\;\hat{s}$(t)^2^ as the product of noise and the spectral subtraction factor $\;|\hat{s}$$(f){|}^{2}=|\hat{n}$(f)|^2^/|**y**(f)|^2^**Formulation:** The Hankel matrix of the enhanced speech signal is formulated from step 6.**Optimization:** The enhanced speech is optimized using the least squares and the minimum variance optimal estimator asHs = ΣV^t^**Conversion:** The enhanced speech signal is converted to text.
**Output:** The text format of the noise removed speech signal with reduced WER.

## 4. Results and Discussion

The proposed model has been tested on a dataset that contains medical speech of about 8.5 h and RAVDESS emotional speech dataset. The speech includes both male and female speeches, with each audio time ranging from 0.2 s to 60 s.

### 4.1. Performance Analysis of Speech Enhancement Algorithms

Various speech enhancement algorithms are present to enhance the speech signal by reducing the noise in the surrounding. The proposed work tested the algorithms such as ISE, sunspace, and NSS for the medical audio dataset, and the findings are discussed in this section.

#### 4.1.1. ISE for Spontaneous Signal

One of the numerous issues that automatic speech recognition systems face is processing spontaneous speech. Spontaneous speech is characterized as utterances that comprise well-formed phrases similar to those found in written texts. Disfluencies (complete pauses, repetitions, false starts, and so on) are the main characteristics of this type of speech, and numerous studies have concentrated on detecting and correcting them.

**Figure 7:**
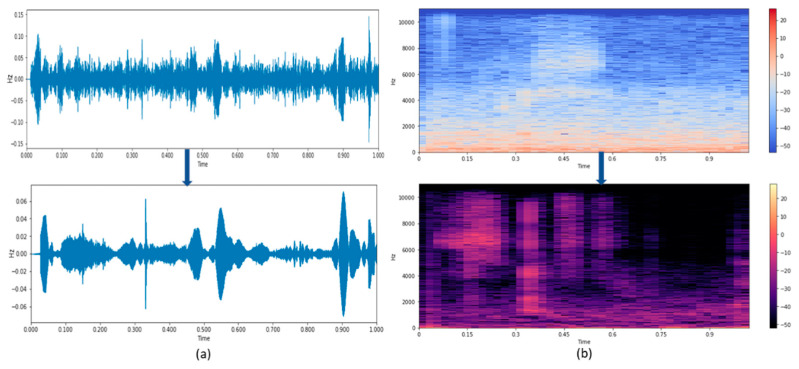
(**a**,**b**) Time waveform and Spectrogram of speech signal using ISE.

The waveform and the spectrogram results produced by the ISE algorithm are shown in Figure 7. Figure 7a,b show a speech signal’s time waveform and spectrogram, respectively. Figure 7a shows how the noisy waveform is getting transferred to noise reduced waveform signal using the ISE algorithm.

Figure 7b represents the spectrogram of a signal that has been enhanced using the ISE. It is visible that there is an enhancement in the noise corrupted speech while using the ISE speech enhancement algorithm. The WER is calculated for the speech before and after removing the noise. The comparison of the WER before and after reducing the noise is given in Table 2.

It is visible from Table 2 that the WER is reduced to a certain extent after using the speech enhancement algorithm ISE, and hence it increases the accuracy of the speech to a minimal extent.

#### 4.1.2. Sub Space Method for Spontaneous Signal

**Figure 8:**
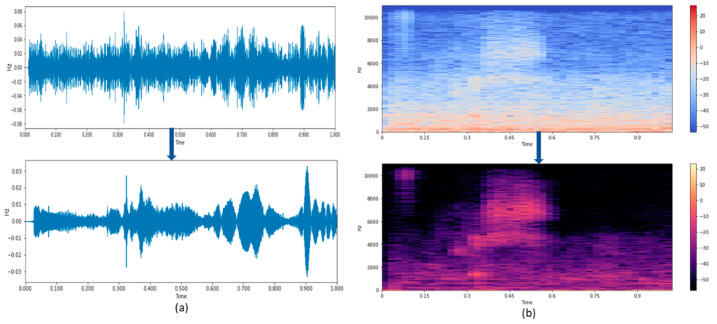
(**a**,**b**) Time waveform and Spectrogram of speech signal using subspace.

The input noise contains the noise from the surroundings, such as the fan and vehicle noises. It is necessary to suppress the external noise to enhance the actual speech to improve its recognition irrespective of its length and complexity. Figure 8a shows the waveform and the spectrogram of the noisy speech signal of length 60 s. When this noisy speech is treated with the subspace method for enhancing the speech, the external noise is removed, and its corresponding spectrogram is shown in Figure 8b. The improved speech can be converted into text for further analysis.

Although the noise removal can be visibly seen in Figure 8b, the quantitative analysis is done by applying Equation (1) in the text of the enhanced speech signal to calculate the word error rate. Table 3 represents the WER calculated, and accuracy of the noise removed signal by using the subspace method.

#### 4.1.3. NSS for Spontaneous Signal

**Figure 9:**
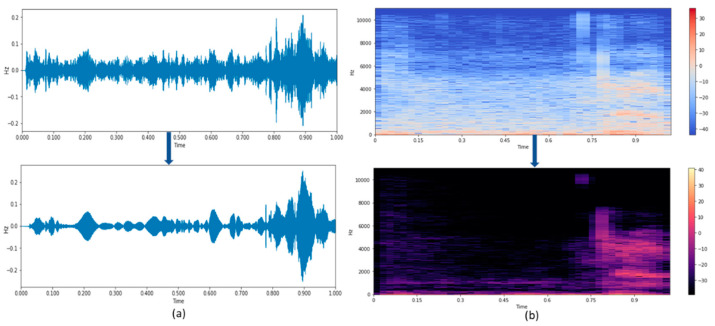
(**a**,**b**) Time waveform and Spectrogram of speech signal using NSS.

Spectral subtraction is one of the most basic and also possibly the most famous speech enhancement methods. Figure 9a shows the time waveform and the spectrogram of the noisy speech signal, where the noise can be any domestic noise in the surroundings. The noise can be effectively removed using the non-linear spectral subtraction, and the corresponding waveform and the spectrogram are shown in Figure 9b. From Figure 9a,b, it is visible that the noise has been eliminated to a greater extent. 

The enhanced speech signal is converted into text using the HMM or the DTW-based text conversion algorithm to find the accuracy level of the enhanced speech signal. Table 4 represents the WER calculated for the enhanced speech signal using the NSS.

Comparing Table 1, Table 2 and Table 3, it can be seen that NSS produces less WER, which makes NSS a more accurate speech enhancement algorithm compared to ISE and subspace methods.

**Figure 10:**
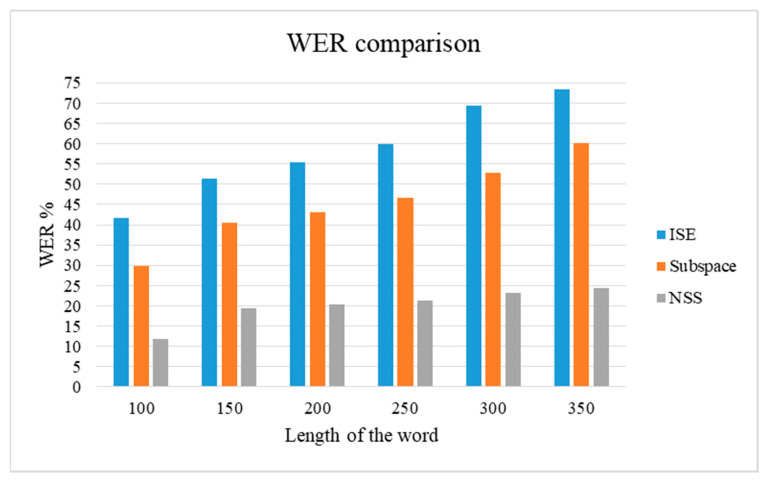
WER comparison of typical SE algorithms.

The WER calculated for the spontaneous signal after enhancing the signal using the NSS, subspace, and ISE are compared and shown in Figure 10. As the number of words increases in the speech, the ISE and subspace produce more WER, whereas the NSS can maintain the accuracy of the speech.

### 4.2. Speech to Text Conversion of Enhanced Speech

The accuracy and efficiency of the speech can be visualized using the time waveform, log transformation, and spectrogram. The text analysis is used to represent the efficiency of the speech with quantitative measurement so that the comparison of speech enhancement becomes accurate.

#### Performance of HMM

The word error rate of a noisy signal and the enhanced speech signal is calculated using the HMM algorithm and is shown in Table 5. It can be seen that the word error rate of a noisy speech is high compared to that of an enhanced speech signal.

### 4.3. Performance Analysis of HSEA

There are various speech enhancement algorithms to reduce the domestic noises recorded during the speech and produce an enhanced signal. When the length of the speech (the number of words involved in the speech) increases, the performance of the algorithms does not seem to be effective as many of them work well with only small words. The enhancement also depends on the complexity of words involved in the speech. Specific applications such as medical speech need more accuracy as the terms involved are complex, and the noise removal should not affect the originality of the words uttered. Hence it is more important to select the best noise removal algorithm for complicated applications.

In the proposed method HSEA, the speech enhancement algorithm NSS and the speech to text conversion algorithm HMM are cascaded to further increase the accuracy of the speech. Table 6 shows the WER of the spontaneous signal with different lengths of words. The WER is calculated for the NSS algorithm without using the HMM speech-to-text conversion method, and the NSS is cascaded with HMM.

**Figure 11:**
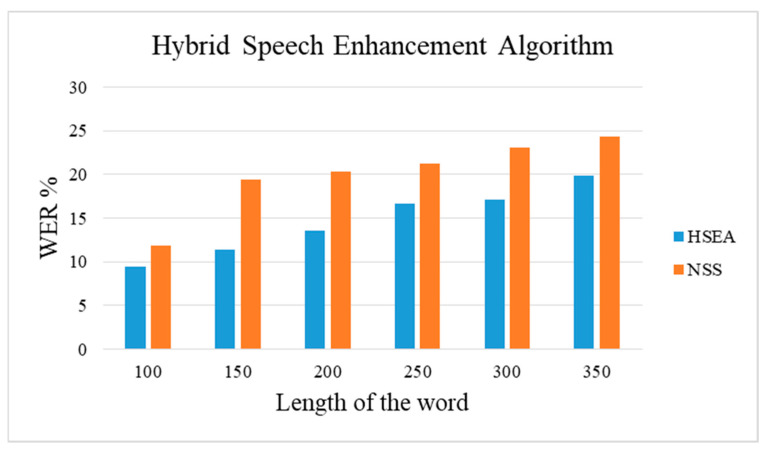
Performance of HSEA for medical speech.

Figure 11 represents the graph for the WER values calculated for the spontaneous signal using the NSS speech enhancement signal with and without cascading it with HMM. Although the length of the spontaneous speech increases, the cascaded hybrid method produces less WER than the stand-alone NSS speech enhancement method.

Table 7 represents the WER calculation for the RAVDESS data with a spontaneous signal. RAVDESS contains audio of different length with both male and female speakers. The WER is calculated using the NSS algorithm and the proposed HSEA method. Compared to Table 6, it is visible that the WER of RAVDESS data is less than that of medical speech. This is because the complexity of medical speech is high compared to that of RAVDESS emotional speech data.

**Figure 12:**
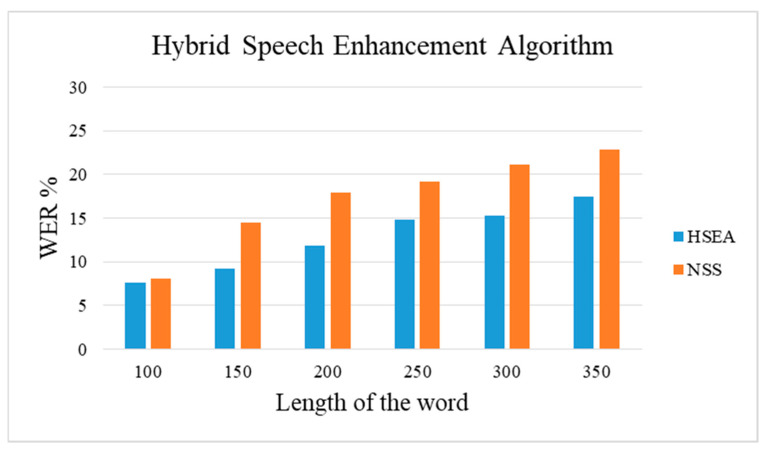
Performance of HSEA for RAVDESS.

Figure 12 describes the performance comparison of HSEA and the NSS algorithm for the RAVDESS dataset with 1440 audio files. It can be seen that RAVDESS has less WER compared to that of the medical speech transcription data as the terms involved in medical speech are complex compared to that of emotional speech. Additionally, HSEA produces less WER compared to that of the NSS algorithm.

## 5. Conclusions

In this study, an HSEA is employed for medical audio data to reduce the word error rate. The entire procedure of the proposed method consists of the NSS and the HMM architecture with the medical data set. The typical NSS algorithm is optimized with least squares and minimum variance and then cascaded with the speech-to-text algorithm HMM to establish the proposed HSEA. In HSEA, the speech is enhanced using the NSS algorithm, and again the enhanced speech is optimized using the optimization criteria of the subspace method. As the double layer of enhancement is performed, HSEA can perform competitively with other typical speech enhancement models such as NSS, ISE, and Subspace. The proposed model has been validated with 6660 medical and 1440 RAVDESS audio data. The validation of the proposed HSEA architecture has proven to achieve maximum accuracy of 90.5% with a minimum word error rate of 9.5% for medical speech transcription and accuracy of 92.4%, the word error rate of 7.6% for the RAVDESS audio data. The validation of the proposed HSEA architecture has proven to achieve maximum accuracy of 90.5% with a minimum word error rate of 9.5%.

The proposed methodology can produce a clear speech signal as the output with reducing word error rate, making it efficient for all kinds of applications involving speech in open space. Despite the advantages, the methodology involves mathematical complexities as the layer of speech purification increases, which remains a limitation of the proposed method. Therefore, future work can concentrate on integrating the layers in such a way as to reduce the computational complexities and to achieve high efficiency.

## Figures and Tables

**Table 1:** Literature survey based on category of techniques.

Techniques	Performance	Advantage(s)	Disadvantage(s)
Spectral subtraction algorithm [2]	Estimating the spectrum of the noise-free signal and subtracting the estimated noisy signal is done to enhance the speech	Can be applied for both stationary and non-stationary noises	Resultant speech contains residual noise
Signal subspace algorithm [3]	Uses Karhunen-Loeve transforms (KLT) or eigenvalue decomposition	Discards the noisy space Can directly use the state-space representation for the system	Realizations done by state-space are not unique
Weiner filter-based algorithm [5]	Mainly used in real time applications	Better performance for noise cancellation	Needs more number of computations
Adaptive Weiner filter-based algorithm [6]	Mainly used in real time applications	Reduced/moderate computational complexity	Mean square error is not always relevant
Deep Auto-Encoder (DAE) algorithm [8]	Deep denoising autoencoders are used to enhance the speech features	Efficient for resonant speech recognition	Mainly used for clean/controlled speech only
Voice Activity Detector (VAD) [10]	Works on the long pause between the words	Can classify the noise even during the pause of the speech	Not efficient for encrypted speech signals
Long Short-Term Memory (LSTM) [11]	A type of RNN, and it can learn long period dependencies	Produces good result in speech recognition	Concentrates only on the size (length) of the speech
Generative Adversarial Networks (GAN) [12]	It is a type of RNN and it constructs clear speech from the noisy speech	Generate audio that looks similar to original audio by eliminating noise	Harder to train
Multiband spectral subtraction algorithm [15]	Inverse filtered reverberation was suppressed by the spectral subtraction	Overcome the distortion, maintaining the quality of speech signal	Not suitable for highly random real-world noise

**Table 2:** WER and its percentage after using ISE.

Length of the Word	WER (%)	Accuracy (%)
100 words	41.6	58.4
150 words	51.5	48.5
200 words	55.5	44.5
250 words	59.9	40.1
300 words	69.5	30.5
350 words	73.4	26.6

**Table 3:** WER and its percentage using subspace method.

Length of the Word	WER (%)	Accuracy (%)
100 words	29.8	60.2
150 words	40.4	49.6
200 words	43.1	46.9
250 words	46.7	43.3
300 words	52.8	37.2
350 words	60.2	29.8

**Table 4:** WER and its percentage using NSS.

Length of the Word	WER (%)	Accuracy (%)
100 words	11.9	88.1
150 words	19.4	80.6
200 words	20.3	79.7
250 words	21.2	78.8
300 words	23.1	76.9
350 words	24.3	75.7

**Table 5:** WER count and percentage with and without noise.

Length of the Word	WER (%) with Noise	WER (%) without Noise
100 words	26.2	21.4
150 words	28.1	23.3
200 words	31.6	28.9
250 words	34.9	32.7
300 words	41.5	39.8
350 words	44.3	41.2

**Table 6:** WER, accuracy using the HSEA and NSS.

Length of the Word	WER (%)	
	HSEA	NSS
100 words	9.5	11.9
150 words	11.4	19.4
200 words	13.6	20.3
250 words	16.7	21.2
300 words	17.1	23.1
350 words	19.9	24.3

**Table 7:** WER, accuracy using the HSEA and NSS for RAVDESS.

Length of the Word	WER (%)	
	HSEA	NSS
100 words	7.6	8.1
150 words	9.2	14.5
200 words	11.9	17.9
250 words	14.8	19.2
300 words	15.3	21.1
350 words	17.5	22.9

## Data Availability

Publicly available datasets were analyzed in this study. This data can be found here: https://www.kaggle.com/paultimothymooney/medical-speech-transcription-and-intent.
